# Artificial Mushroom Sponge Structure for Highly Efficient and Inexpensive Cold‐Water Steam Generation

**DOI:** 10.1002/gch2.201800035

**Published:** 2018-10-15

**Authors:** Xiujun Gao, Haihang Lan, Songru Li, Xubing Lu, Min Zeng, Xingsen Gao, Qianming Wang, Guofu Zhou, Jun‐Ming Liu, Michael J. Naughton, Krzysztof Kempa, Jinwei Gao

**Affiliations:** ^1^ Institute for Advanced Materials South China Academy of Advanced Optoelectronics and Guangdong Provincial Key Laboratory of Quantum Engineering and Quantum Materials South China Normal University Guangzhou 510006 P. R. China; ^2^ School of Chemistry and Environment South China Normal University Guangzhou 510006 P. R. China; ^3^ Electronic Paper Displays Institute South China Academy of Advanced Optoelectronics South China Normal University Guangzhou 510006 P. R. China; ^4^ Laboratory of Solid State Microstructures Nanjing University Nanjing 210093 P. R. China; ^5^ Department of Physics Boston College Chestnut Hill MA 02467 USA

**Keywords:** artificial mushrooms, PVA, solar vapor conversion, soot

## Abstract

A bioinspired structure of an artificial mushroom, made of the common polyvinyl alcohol sponge coated with charcoal, is fabricated, with high efficiency in generating cold water steam of ≈73% under 1 sun illumination, due to very high light absorption, efficient water supply, and low heat loss. In addition, the structure is very inexpensive, and thus ideal for applications in portable cold (boil‐free) steam generators for water purification and desalination.

Excessive energy consumption and fresh water scarcity are recognized as two main components of increasingly threatening global issues for sustainable social development.^1^ Generating fresh water using solar energy is a highly promising strategy to relieve stresses associated with water shortage.^2^, ^3^, ^4^ In this context, solar‐induced, cold water steam generation, a green, clean, sustainable, inexhaustible, and renewable technology is becoming a viable research area.^5^, ^6^, ^7^, ^8^, ^9^, ^10^, ^11^, ^12^, ^13^, ^14^, ^15^, ^16^, ^17^ Early solutions to solar water desalination employed solid absorbers heating bulk water directly, under high optical concentration. These suffered from large optical and heat loss, and consequently low efficiency.^18^, ^19^, ^20^, ^21^, ^22^, ^23^, ^24^ Various strongly light absorbing materials and structures have been explored and applied in such structures, including graphene, graphene oxide, metallic mesh structures, semiconductor nanoparticles, and plasmonic structures.^11^, ^25^, ^26^, ^27^, ^28^ While such materials mostly suffer from relatively high cost, and limitation in their practical applications.

Modern solutions are aimed at enhancing solar absorption efficiency by reduction of thermal losses. In an evaporation system, the heat loss involves three components, radiative heat loss to the ambient, convective and conductive heat loss to the underlying water. This can be achieved in specially designed all‐in‐one structures, which concentrate and maximize solar absorption only at the surface exposed to light, while maintaining high water transport speed, and large temperature gradient away from the surface. Such all‐in‐one structures include a 3D‐printed evaporator,^29^ a bilayer hybrid biofoam based on bacterial nanocellulose and reduced graphene oxide,^10^ a structure based on graphite oxide,^30^ a device with confined 2D water path,^15^ and a vertically aligned graphene sheet membrane.^31^ However, these solutions are either expensive, complicated to fabricate, or have a very short lifetime to be practical. Recently, increasing low‐cost system referring to bioinspired and common material solutions including structures based on paper,^32^ wood,^26^, ^33^, ^34^ and carbonized mushrooms^35^ have been proposed to address these problems. Mushroom structure has been confirmed that its large surface area to projected area ratio minimizes the loss from radiation and convection. Herein, for the first time, we introduce a common material, polyvinyl alcohol (PVA) sponge (cut from gray‐dyed PVA‐based commercial mop heads) which behaves of several intrinsic merits for solar steam generation system, such as extraordinary hydrophilic behavior and water absorption capacity ensuring enough and quick water supply for evaporation system, high photothermal activity rapidly appearing higher surface temperature under solar illumination, ultralow thermal conductivity leading to good heat confinement within the evaporative interface by minimizing the heat transfer to bulk water, and improving the overall efficiency of the solar steam generation. Based on those intrinsic properties of PVA, inspired by the unique structure of mushroom and plants in nature, we eventually developed a PVA‐soot artificial mushroom structure for a high efficient solar steam generation system (≈73% under 1 sun illumination), by using a very simple and cheap processing of candle smoking and transferring.

**Figure** **1**a shows a schematic of our artificial mushroom structure, including a cap (for light absorption) and a stem (for water supply). Upward flow of water in the stem, from the reservoir below to the cap (and throughout the cap) occurs by capillary motion (blue arrow in Figure 1a). The cap is coated with an absorber material to facilitate large light absorption‐based heating of the cap. Reducing the stem diameter reduces the heat flow between the cap and the water below (red arrow in Figure 1a), which is advantageous for localized surface heating of the cap, but this also reduces water flow, which could eventually reduce the evaporation rate. To prevent the latter effect, one must assure very strong capillary action inside the stem. A simple analysis quantifies the action of this structure: solar radiation delivers energy to the cap, which simultaneously loses some of it due to thermal transport via the stem to the water in the reservoir below. The net delivered energy heats up the cap, thus

(1) $$d\rho_{w}c_{w}\Delta T\approx P_{\mathrm{rad}}-\kappa_{w}\Delta T(A_{s}/A_{c})$$

where *A*_s_ is the cross‐sectional area of the stem, *A*_c_ is the top surface area of the cap, *d* is the cap thickness, ρ_w_ is the cap material density, *c*_w_ is the specific heat of the cap material, and κ_w_ is the thermal conductivity of the stem. Here, the cap material refers to wet sponge after fully water absorbing. Then ∆*T* is the temperature difference between the cap and the water in the reservoir below, and *P*_rad_ is the power density absorbed in the cap, which is proportional to the absorbance of the coating. From Equation (1), the temperature difference is

(2) $$\Delta T\approx \frac{P_{\mathrm{rad}}}{d\rho_{w}c_{w}+\kappa_{w}\left(A_{s}/A_{c}\right)}$$

shape maximized for small aspect ratio, *A*_s_/*A*_c_ << 1. The evaporation rate ξ was calculated by Penner,^36^ and can be fitted to the following dependence

(3) $$\xi \approx A_{c}\rho_{w}\xi_{0}e^{\alpha \Delta T}$$

where *ξ_0_* = 0.0336 cm s^−1^, and α = 0.07311 K^−1^. Clearly, maximizing ∆*T* is the key to maximum performance of the evaporation device. However, the evaporation rate ξ must be matched by the water flow rate ς in the stem, given by

(4) $$\varsigma =A_{s}\rho_{w}\nu_{\mathrm{capil}}$$

where *v*_capil_ is the speed of liquid in the stem due to capillary action. The matching condition requires that ς ≥ ξ, and therefore

(5) $$\nu_{\mathrm{capil}}\geq \left(\frac{A_{c}}{A_{s}}\right)\;\xi_{0}e^{\alpha \Delta T}$$

**Figure 1:**
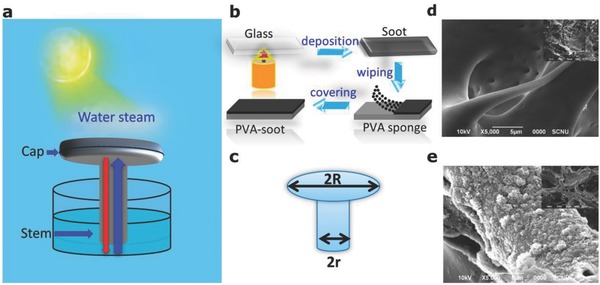
a) Schematic of the artificial mushroom for cold water steam generation. The blue arrow symbolizes water flow by capillary action from the container to the cap, and the red arrow symbolizes the deleterious heat transport from the heated cap to the water container. b) Coating procedures of the cap with soot, deposited from the carbonized smoke from a candle. c) Dimensions of the artificial mushroom. d) SEM images of the bare, and e) coated with soot PVA sponge.

Thus, finally, maximizing evaporation requires maximizing ∆*T* for *A_s_*/*A*_c_ << 1, while satisfying inequality (5).

We have investigated various materials for the stem and the cap. Specifically for the stem, we tested wood, paper, PVA sponge, expanded polyethylene (EPE), and polyurethane (PU). **Figure** **2**a shows that the PVA sponge has the best water absorption capacity (ratio of weights after to before soaking). This suggests strong capillary action, and indeed PVA sponge exhibits hydrophilic behavior with a water contact angle approaching 0° (Figure S1, Supporting Information), enabling enough and quick water supply for evaporation system from stem to cap in time.

**Figure 2:**
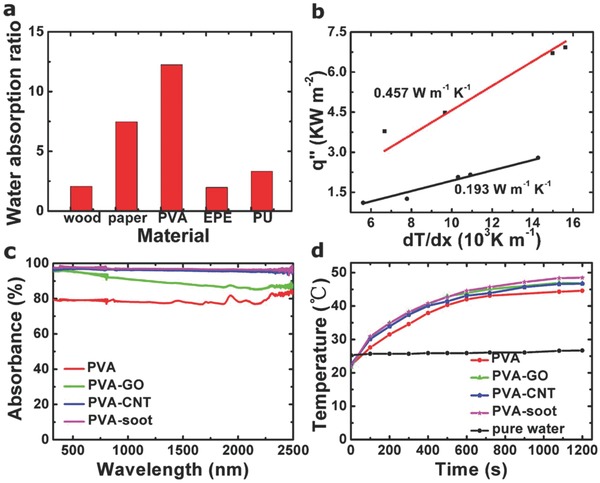
a) Water absorption capacity (ratio of weight before and after soaking) of various tested supporting materials. b) Thermal conductivity κ_w_ curves of the PVA sponge in the dry (black) and wet (red) conditions. c) Light absorbance versus wavelength for the PVA cap with various coatings. d) The surface temperature versus time for our artificial mushroom (with (*r*/*R*)^2^ = 1.5%) with various cap coatings, and for the water in the reservoir. Standard AM1.5 irradiation conditions (1 sun) was used.

IR temperature photographs of different samples were provided for comparison after 10 min solar illumination under 1 sun. Figure S2 (Supporting Information) demonstrated the temperature of PVA sponge rapidly increases from ≈24 to ≈84 °C (Δ*T* = 60 °C), wood reaches to 56 °C (Δ*T* = 32 °C), airlaid paper reaches to 33 °C (Δ*T* = 9 °C), EPE sponge reaches to 32 °C (Δ*T* = 8 °C), and PU sponge reaches to 46 °C (Δ*T* = 22 °C). It is obvious that PVA sponge has the highest surface temperature when illuminated, suggesting a highly optical absorption and photothermal activity of the PVA sponge.

Figure 2b shows that the PVA sponge has a thermal conductivity κ_w_ in wet conditions, which is lower than other materials reported recently in the field of thermal steam generation,^26^, ^33^, ^35^ which leads to the confinement of heat to evaporative interface by minimizing the heat transfer to bulk water and improve the overall efficiency of the solar steam generation. As well, the thermal conductivity of dry PVA sponge is also very low, shown in Figure 2b. All thermal conductivity measurements were conducted by a steady‐state method, shown in Figure S3 (Supporting Information) and the Experimental Section. In addition, wet PVA sponge possesses excellent mechanical properties, superior to natural/carbonized mushroom,^35^ allowing it to be easily bent, stretched, and twisted (Figure S4, Supporting Information), so that they can be washed thoroughly to clean the salt/contaminants at any time even in practical full desalination system applications. Based on these results, it comes to the conclusion that PVA sponge has numerous properties, which makes it great potential candidate for high‐performance solar steam generation. So we chose this material for the structure of our artificial mushroom.

To assure good light absorption, we have tested several coatings of the cap, including graphite oxide (GO), carbon nanotubes (CNT), and soot. Figure 2c shows that the highest light absorbance (visible to infrared range) is achieved with PVA sponge uniformly coated with soot (the least expensive coating) or CNT (98%), followed by PVA sponge uniformly coated with GO (87–98%) and bare PVA sponge (≈80%). These results have been confirmed by Fourier transform infrared (FTIR) and Raman spectral characterizations (Figures S5 and S6, Supporting Information). Figure 2d shows temperature variations (vs time) induced by exposure to light (AM1.5), for PVA mushroom coated with various absorbers, as well as the temperature of the water in the reservoir. Again, soot provides the best performance, with a maximum temperature of about 48.5 °C, followed by GO or CNT (≈46.5 °C), and no coating (≈44.5 °C). The maximum temperature of the water in the reservoir is ≈27 °C.

Based on these results, we chose soot as the cap coating material to fabricate the solar vapor conversion system. The process is schematically shown in Figure 1b, which simply includes two steps, generating soot by directly exposing a piece of glass to the candle smoke, followed by transferring soot into porous sponge through directly wiping and sucking soot particle into porous sponge. The process is much simpler than that of mushroom carbonized treatment.^35^ Optical images of samples with and without a soot layer are shown in Figure S7 (Supporting Information). A sketch of the mushroom structure is shown in Figure 1c with definitions of *R*, the diameter of the cap and *r*, the diameter of the stem. Specific parameters are given in Figure S8 and Table S1 (Supporting Information). Figure S9 (Supporting Information) shows the lightweight character of the PVA sponge, potentially of benefit for a floating solar vapor conversion system. Microscopic structures of the PVA sponge before and after soot deposition are shown in the scanning electron microscopy (SEM) images of Figure 1d,e, respectively. The PVA sponge is a synthetic porous material, advantageous for rapid water transport and steam escape (see Figure S10, Supporting Information). After soot deposition, most of the soot particles were sucked into the PVA pores and provided a large interface for efficient, solar conversion of water to vapor (Figure 1e and Figure S11, Supporting Information).

To explore an optimal structure for solar conversion to vapor, we studied the performance of PVA samples with different area ratios, *S* = *A*_s_/*A*_c_, as shown in **Figure** **3**a. PVA samples *S*_0_–*S*_5_ were prepared with values of 100, 29, 57, 33, 11, and 1.5%, respectively. The corresponding mass changes during evaporation are shown in Figure 3b for the samples with different *S* values, and the evaporation rate (and conversion efficiency) are shown in Figure 3c. The average evaporation rate of pure water in an ambient dark environment is 0.286 kg m^−2^ h^−1^, shown in Figure S12 (Supporting Information), which is subtracted from all measured evaporation rates to eliminate the effect of natural water evaporation. These evaporation rates with only bare PVA sponge are higher than most previously reported results.^33^, ^37^ This result confirms PVA's speciality and several merits in above mentioned details. The monotonically increasing ξ for vanishing stem diameter *r* indicates that inequality (5) is satisfied, that is, capillary action remains strong (ς ≥ ξ) for all measured artificial mushrooms. The corresponding solar thermal conversion efficiencies, calculated according to ref. 38, are also shown in Figure 3c. These demonstrate that the capillary action in the PVA sponge stem is not a bottleneck and remains highly efficient even at the smallest stem radius (*S*_5_ = 1.5%). Figure 3d shows the temperature variation of water in the reservoir for mushrooms with *S*_0_ and *S*_5_, corresponding to the maximum and minimum area ratios. As expected, narrow stem *S*_5_ reduces heat transfer between the cap and the water in the reservoir and contributes to heat localization within the cap. Compared with natural/carbonized mushroom,^35^ artificial sponge mushroom can be easily geometrically tunable and optimized in practical application.

**Figure 3:**
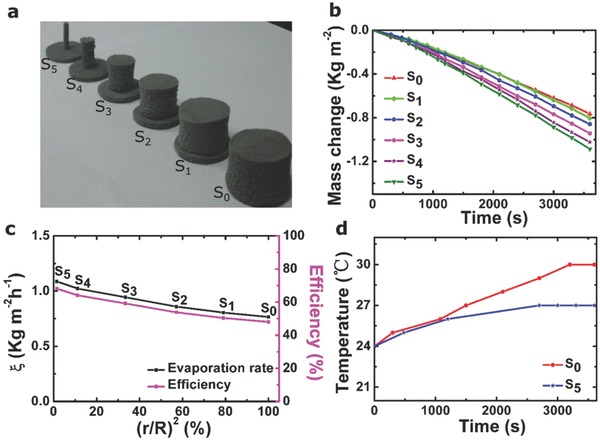
a) Optical images for PVA sponges with different area ratios. b) Water mass change during light illumination for bare PVA mushroom samples, with different area ratios. c) Evaporation rate ξ and solar‐to‐vapor conversion efficiency of the artificial mushrooms based on bare PVA sponge, versus the area ratio (*A*_s_/*A*_c_). d) Temperature versus time for water in the reservoir during the evaporation process for the samples with two area ratios.

We chose the optimal mushroom structure *S*_5_ for coating experiments. **Figure** **4**a shows IR images of the final evaporation system of PVA (S_5_)‐soot coating at various illumination times. This evaporation system shows good performance in that the surface temperature quickly increases from 23 to 45 °C (within 10 min) and also good heating uniformity. The evaporation setup is shown in Figure S13 (Supporting Information). Obvious under solar illumination, water steam generates from the evaporation system, and vice versa, there is no vapor generation in a dark environment (Figure S14, Supporting Information). Figure 4b shows the mass change after coating with various light absorbing materials (GO, CNT, and soot). Compared to the bare PVA sponge, PVA with coatings shows good solar absorption capability and subsequent water evaporation rate. The inset of Figure 4b clearly shows the difference between the bare PVA and the coated systems. All of the coated systems show almost the same performance in mass change, but the soot coating is expected to have the lowest cost compared to graphene and graphene oxide materials. This priority is further shown in Figure 4c, a comparison of the conversion efficiencies and price for our artificial mushroom system and references under 1 sun illumination. While the efficiency of our structure (72.4%) is comparable to that in other reported results, it is not the highest.^13^, ^15^, ^25^, ^31^, ^33^, ^39^ However, our artificial mushroom system should be, by far, the least expensive, and thus the most practical devices than others (a rough cost comparison for different devices in Table S2, Supporting Information). Thus, it is suitable for widespread portable implementation even in the developing world such as a poor village to generate fresh water from the dirty water. Note that we did not precisely calculate the cost of different materials, only roughly classified those into two series, low price (PVA + soot, and wood) and high price (graphene and graphene oxide coatings, and noble metal nanoparticle floating systems).

**Figure 4:**
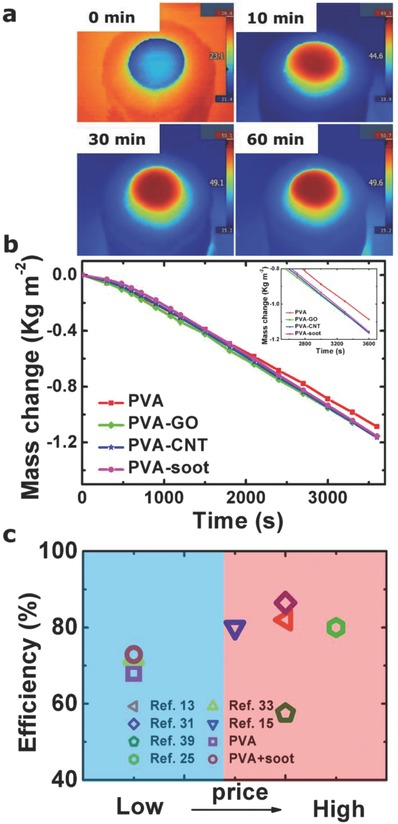
a) IR images showing the surface temperature at four different time‐point. b) Water mass change versus time for artificial mushroom structure with and without coatings. c) Conversion efficiencies (under 1 sun illumination) of various cold evaporator structures versus cost.

In conclusion, we have demonstrated a cost‐effective solar steam generation system, with a bioinspired structure of an artificial mushroom made of common PVA sponge, coated with soot. The best solar‐to‐vapor efficiency of the PVA + soot artificial mushroom structure is ≈73% under 1 sun, which benefits from the good light absorption material (soot), efficient water supply (PVA sponge), and excellent thermal management with minimized heat loss (i.e., the low thermal conductivity of PVA sponge and the artificial mushroom structure). In addition, the materials used are quite common, inexpensive, robust, and the processing is easily scalable for large size R2R production, thus ideal for practical applications in steam generators for water purification and desalination.

## Experimental Section

*Preparation of the PVA Sponge*: Gray‐dyed PVA‐based commercial mop heads, a daily cleaning tool, were purchased from a supermarket. These were soaked in deionized (DI) water for several minutes, cleaned, and then dried in an electric oven at 80 °C for 48 h. Dried PVA mopheads were cut into various desired mushroom shapes. All PVA sponge in this paper were directly cut from gray‐dyed PVA‐based commercial mop heads.

*Preparation of the PVA‐Coating System*: Graphene oxide sheets were purchased from Sigma‐Aldrich (Cat. No. 763713). The PVA‐GO composite was prepared by drop‐casting aqueous GO solution (0.3 wt%) onto the top surface of a cap and dried. 480 mg CNT (Alfa Aesar) was dispersed in 10 mL DI water and treated with sonication for 2 h to gain a homogenous solution. To obtain the PVA‐CNT composite, aqueous CNT solution was drop cast on the surface of the PVA sponge and dried. The glass plate was smoked over a candle flame for ≈1 min. Then, the soot was transferred from the glass to the surface of wet PVA sponge cap.

*Characterization*: The morphology and structure of the samples were characterized by a SEM system (JEOL JCM‐5700). FTIR spectra were obtained on a Vertex 70/80 spectrometer. The optical transmittance and reflectance spectra of the surface of PVA and PVA‐based composites were measured in the range of 300–2500 nm with a Lambda 950 UV–vis spectrometer attached to an integrating sphere. The absorption efficiency was then calculated by *A* = 1 − *R* − *T*, where *R* and *T* are the reflection and transmission efficiency, respectively. The Raman spectra were obtained using a Renishaw inVia confocal Raman spectrometer mounted on a Leica microscope with a 50× objective and a 532 nm wavelength diode laser as an illumination source. A Fluke Ti 100 thermal imager was used to take infrared photographs. The water mass change through evaporation was measured using an electronic mass balance with an accuracy of 0.001 g. The steam generation experiments were conducted in the lab using a solar simulator (CEL‐HXF300) with an optical filter for the standard AM 1.5 G spectrum. The optical detector in an FZ‐A irradiatometer is smaller in area than the solar receiver, and the maximum measured solar flux is regarded as the actual constant solar flux for the efficiency measurements.

*Thermal Conductivity Measurements*: Thermal conductivities were measured using a steady‐state method with homemade test equipment. The thermal conductivity of PVA sponge in the dry and wet state was measured by sandwiching the materials between two glass slides. The sandwich structure was placed on a room temperature plate with hot water on the top side of glass. The temperature distribution along the cross section of the sandwich structure was monitored using the Fluke IR camera. The calculation of thermal conductivity was based on the assumptions that the sample and the glass slides were experiencing the same heat flux, and the emissivity coefficients of the sample and glass slide were 0.9.

*Solar Steam Generation Experiments*: A specified PVA/PVA‐coating system was placed in a punched expandable polyethylene foam (thermal conductivity ≈ 0.027 W m^−1^ K^−1^) with the stem inside the hole, and the entire structure was allowed to float on the surface of DI water in a quartz beaker with only the bottom side of the stem in direct contact with bulk water. The samples were irradiated by a solar simulator with only the top surface of the system cap directly illuminated. The optical concentration was constant at 1 kW m^−2^, and the evaporation rates were measured for 60 min under steady‐state conditions. Both the weight loss and surface temperatures over the entire process were recorded using an electronic mass balance and IR camera, respectively. The temperature of the water bath was measured by thermocouples. The room temperature was ≈23 °C and the ambient humidity was ≈40%. Solar evaporation system of PVA‐based artificial mushroom sponge structure is shown in Figure S15 (Supporting Information). The energy conversion from solar illumination to thermal energy for steam generation was simply calculated from the evaporation amount of water. In the calculation of efficiencies in this work, only the phase‐change enthalpy is considered, more detailed arithmetic for calculating efficiency is provided in the Supporting Information, and all the data in the figures are average values of several samples. The average evaporation rate of pure water in the dark is subtracted from all the measured evaporation rates to eliminate the effect of natural water evaporation.

## Conflict of Interest

The authors declare no conflict of interest.

## Supporting information

Supplementary
