# Supplementary material for: Artificial Mushroom Sponge Structure for Highly Efficient and Inexpensive Cold‐Water Steam Generation
Source: Glob Chall. 2018 Oct 15;2(12):1800035. doi: 10.1002/gch2.201800035 (PMC6607233; doi:10.1002/gch2.201800035)
Supplement: Supplementary file 1 — Supplementary [file GCH2-2-1800035-s001.pdf]

Copyright WILEY-VCH Verlag GmbH & Co. KGaA, 69469 Weinheim, Germany, 2018.

Global Challenges
Open Access

Supporting Information

for *Global Challenges,* DOI: 10.1002/gch2.201800035

Artificial Mushroom Sponge Structure for Highly Efficient and Inexpensive Cold-Water Steam Generation

*Xiujun Gao, Haihang Lan, Songru Li, Xubing Lu, Min Zeng, Xingsen Gao, Qianming Wang, Guofu Zhou, Jun-Ming Liu, Michael J. Naughton, Krzysztof Kempa, and Jinwei Gao**

Copyright WILEY-VCH Verlag GmbH & Co. KGaA, 69469 Weinheim, Germany, 2016.

Supporting Information

Artificial mushroom sponge structure for highly efficient and inexpensive cold-water steam generation

*Xiujun Gao, Haihang Lan, Songru Li, Xubing Lu, Min Zeng, Xingsen Gao, Qianming Wang, Guofu Zhou, Jun-Ming Liu, Michael J. Naughton, Krzysztof Kempa, and Jinwei Gao**

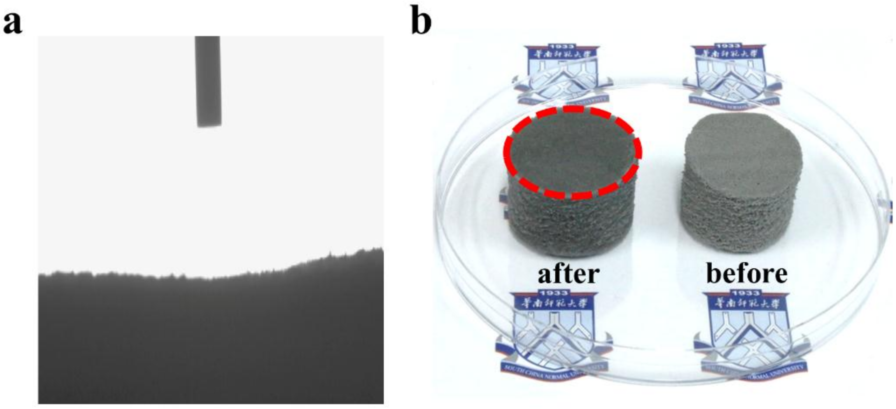

Figure S1. The contact angle of PVA sponge (a) and photographs (b) of PVA sponge before and after water absorption. PVA sponge is directly cut from gray-dyed PVA-based commercial mop heads. The red line in (b) shows that the surface of PVA sponge is filled with water indicating its hydrophilic behavior.

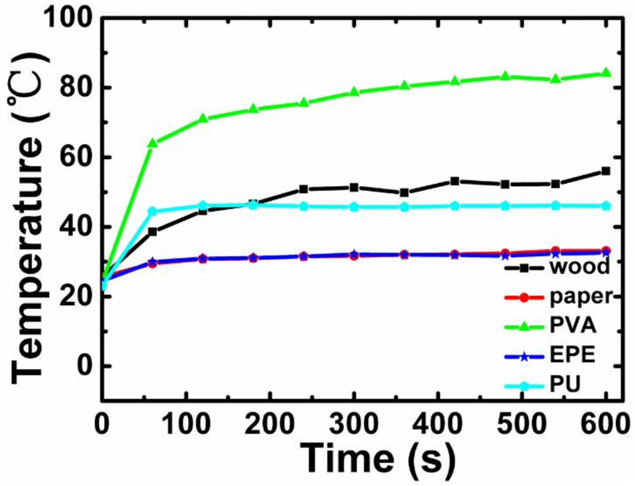

Figure S2. The surface temperature variation of five common materials such as wood, paper, PVA sponge, EPE, and PU sponge after 10 mins solar illumination under one sun.

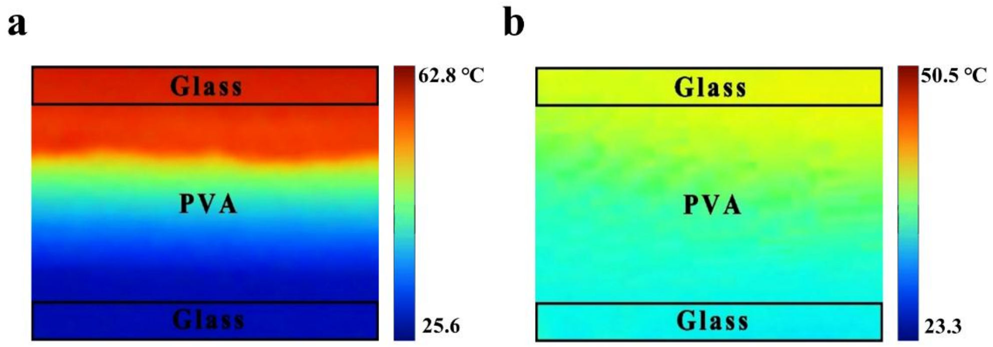

Figure S3. Infrared images of dry PVA sponge (a) and wet PVA sponge (b) in the process of measuring thermal conductivity.

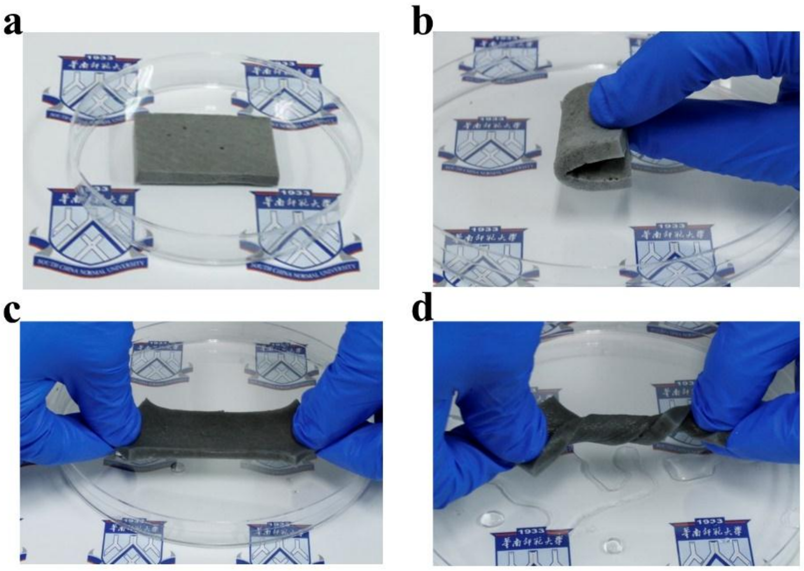

Figure S4. The various flexible properties of wet PVA sponge such as folding (b), stretchable (c), and enable twisting (d) after fast water absorption of dry sponge (a).

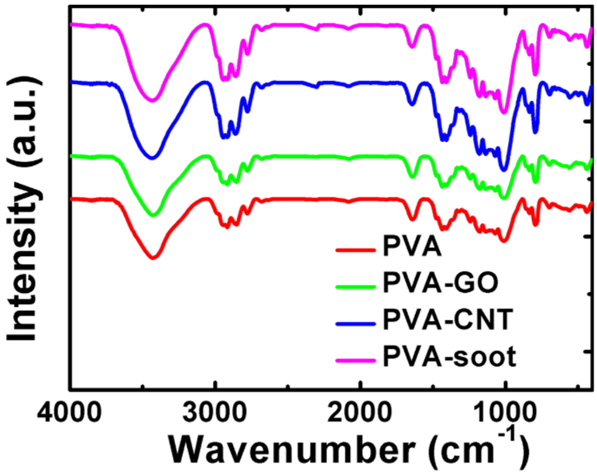

Figure S5. FTIR spectrum of PVA-based composites.

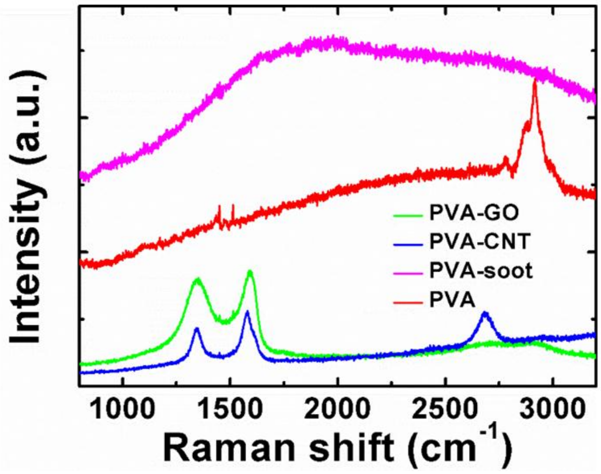

Figure S6. Raman spectra of PVA sponge, PVA-GO, PVA-CNT, and PVA-soot composite.

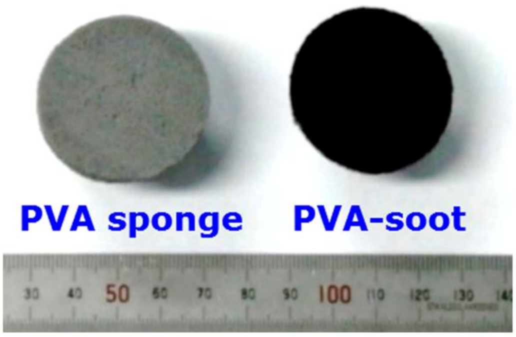

Figure S7. Optical images of the PVA sponge (left) and PVA coated with soot (right).

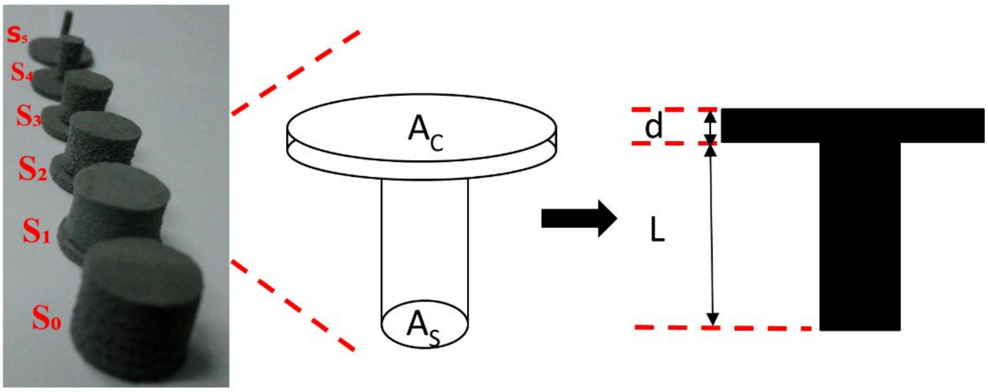

Figure S8. Mushroom structure composition and parameter definition.

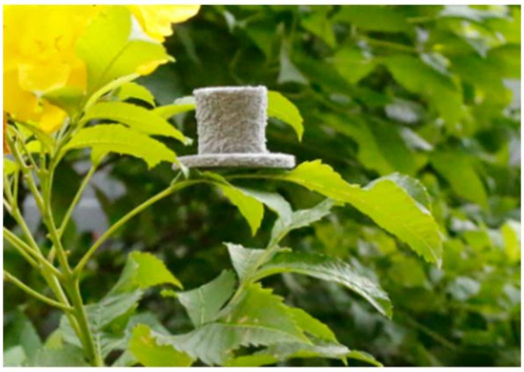

Figure S9. The light weight character of a PVA sponge.

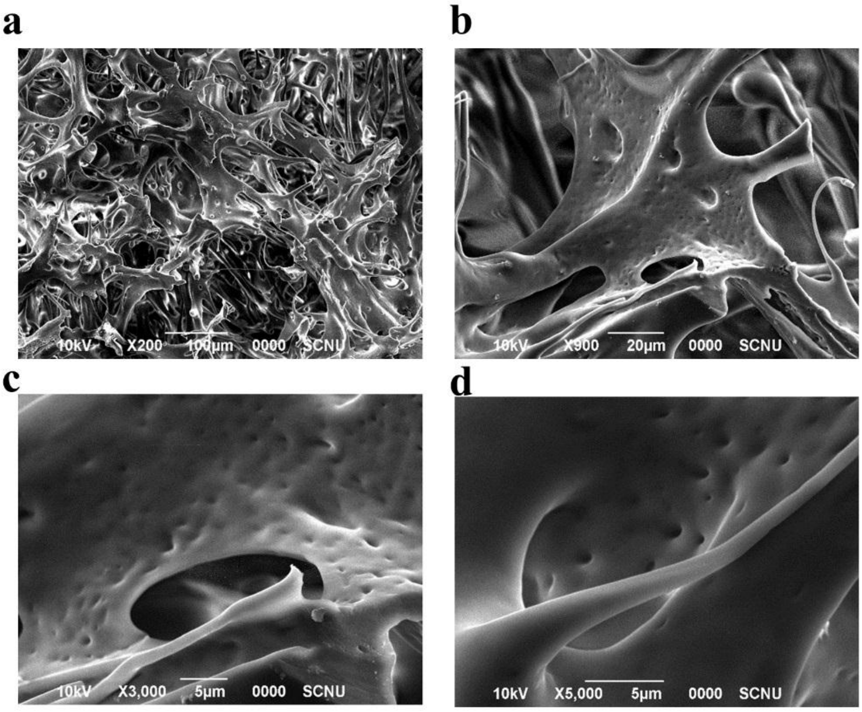

Figure S10. SEM images of PVA sponge under different magnification (a) 200, (b) 900, (c) 3000, (d) 5000.

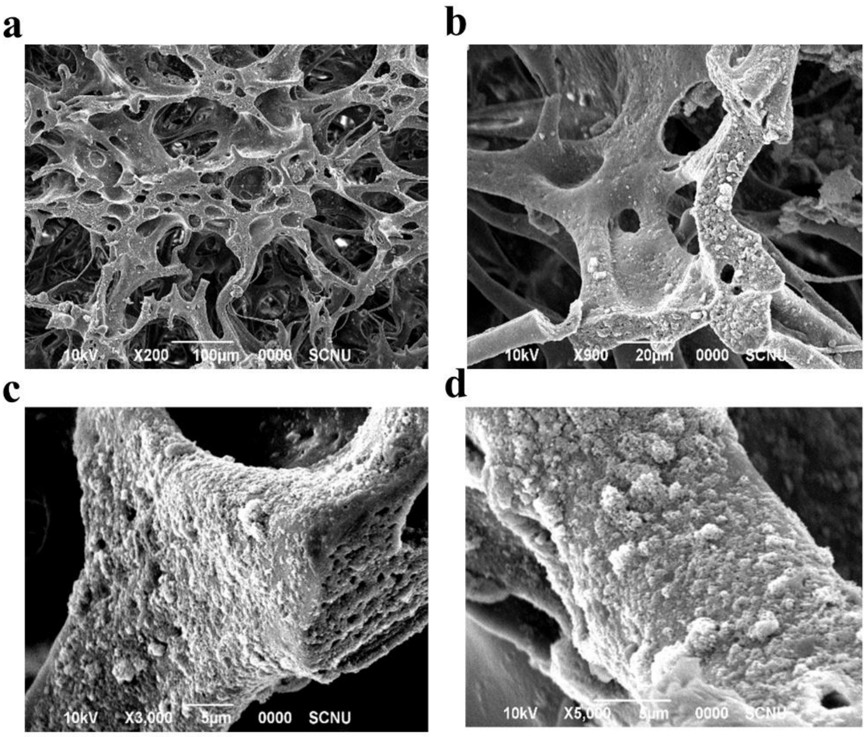

Figure S11. SEM images of PVA-soot composite under different magnification (a) 200, (b) 900, (c) 3000, (d) 5000.

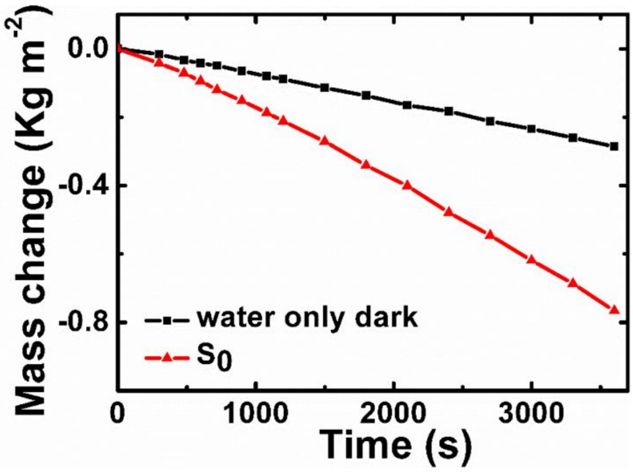

Figure S12. Mass change of pure water in only dark environment and regular PVA sponge (S_0 sample) under 1 sun.

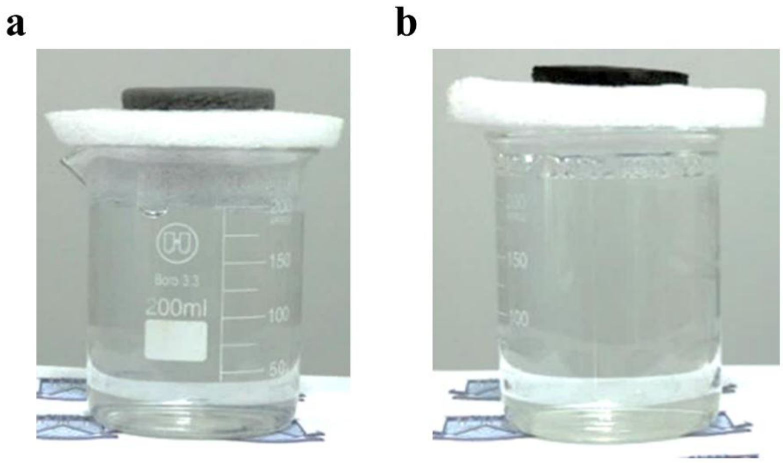

Figure S13. PVA (a) and PVA-coating (b) systems for solar steam generation.

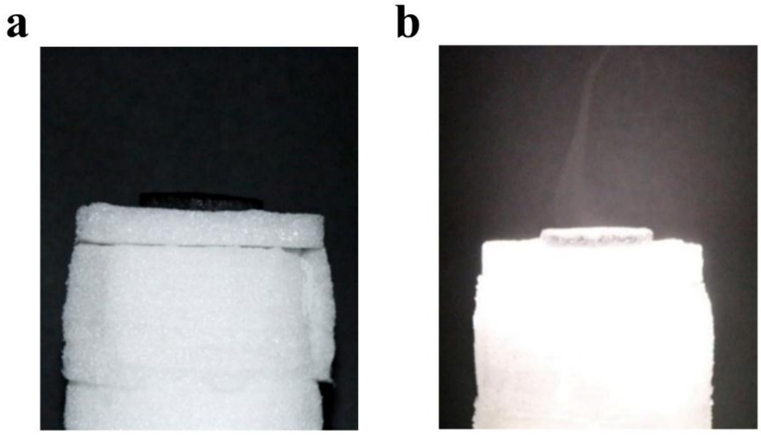

Figure S14. Obvious steam product of PVA-soot device upon irradiation (b) for solar steam generation compared with in dark environment (a).

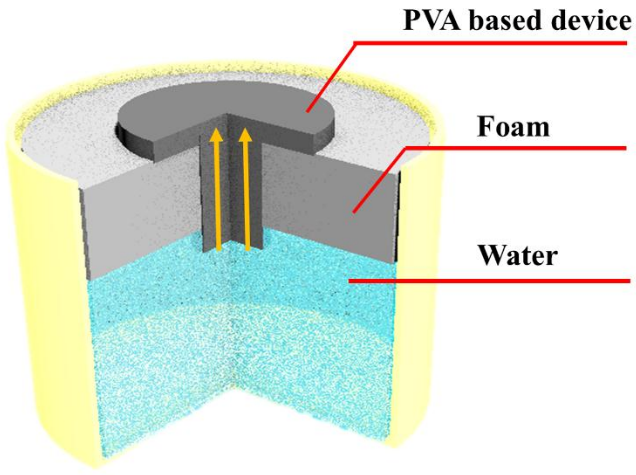

Figure S15. Solar evaporation system of PVA-based artificial mushroom sponge structure

The energy conversion from solar illumination to thermal energy for steam generation was simply calculated from the evaporation amount of water. The solar thermal conversion efficiency (η) is given by:[1]

$$\eta = \frac{\dot{m}hLV}{I} \qquad (1)$$

where η is solar thermal conversion efficiency, $\dot{m}$ is the evaporation rate, h_{LV} is the total enthalpy of sensible heat (315 J g^{-1}, from ca. 25 to 100 °C with specific heat of 4.2 J g K^{-1}) and phase change of liquid to water (2256 J g^{-1}), and I is the solar illumination energy. In the calculation of efficiencies in our work, only the phase-change enthalpy is considered, and all the data in the figures are average values of several samples. The average evaporation rate of pure water in the dark conditions is subtracted from all the measured evaporation rates to eliminate the effect of natural water evaporation.

Table S1. Specific parameters of six PVA mushroom samples (S0~S5).

sample	size(mm)	S_0	S_1	S_2	S_3	S_4	S_5
dry	height	21+4	21+4	21+4	21+4	21+4	21+4
	diameter	40	37	32	23	13	5
wet	height	26+5	26+5	26+5	26+5	26+5	26+5
	diameter	45	40	34	26	15	5.5
	Area ratio (As/Ac)	100%	79%	57%	33%	11%	1.5%

Table S2. Rough cost comparison between our samples and other state-of-the-art solar steam generators (per one device).

NO.	Solar steam generator	Price for material	Price for processing	Total cost	Ref.
1	PVA artificial mushroom	10	5	15	Our work

2	PVA + soot artificial mushroom	10	5	15	Our work
3	CNT film + silica	100	20	120	2
4	graphene sheets membrane	100	50	150	3
5	aluminium nanoparticles	50	30	80	4
6	nanoporous graphene	95	20	115	5
7	flame treated wood	5	20	25	6
8	foldable graphene oxide film-based device	75	15	90	7

[1] H. Ghasemi, G. Ni, A. M. Marconnet, J. Loomis, S. Yerci, N. Miljkovic, G. Chen, *Nat. Commun.* **2014**, 5,7.

[2] Y. C. Wang, L. B. Zhang, P. Wang, *ACS Sustainable. Chem. Eng.* **2016**, 4, 1223.

[3] P. P. Zhang, J. Li, L. X. Lv, Y. Zhao, L. T. Qu, *ACS nano* **2017**, 11, 5087.

[4] L. Zhou, Y. L. Tan, J. Y. Wang, W. C. Xu, Y. Yuan, W. S. Cai, S. N. Zhu, J. Zhu, *Nat. Photonics* **2016**, 10, 393.

[5] Y. Ito, Y. Tanabe, J. H. Han, T. Fujita, K. Tanigaki, M. W. Chen, *Adv. Mater.* **2015**, 27, 4302.

[6] G. B. Xue, K. Liu, Q. Chen, P. H. Yang, J. Li, T. P. Ding, J. J. Duan, B. Qi, J. Zhou, *ACS Appl. Mater. Interfaces* **2017**, 9, 15052.

[7] X. Q. Li, W. C. Xu, M. Y. Tang, L. Zhou, B. Zhu, S. N. Zhu, J. Zhu, *Proc. Natl. Acad. Sci. USA* **2016**, 113, 13953.
